# Case Report: Functional and Symptomatic Improvement With Cariprazine in Various Psychiatric Patients: A Case Series

**DOI:** 10.3389/fpsyt.2022.878889

**Published:** 2022-08-01

**Authors:** Tommaso Vannucchi, Costanza Taddeucci, Lorenzo Tatini

**Affiliations:** Functional Unit of Adults Mental Health, Mental Health Department, Prato, Italy

**Keywords:** partial agonist, cariprazine, schizophrenia, drug abuse, bipolar disorder, cocaine-seeking relapse, antipsychotic

## Abstract

Cariprazine is a third-generation antipsychotic medication approved for the treatment of schizophrenia and bipolar disorder, with unique pharmacodynamic and pharmacokinetic properties. In this case series, the functional and symptomatic improvement of three patients who had been diagnosed with different psychiatric disorders and who exhibited various symptoms from psychotic to mood symptoms is described. The first case is about a young male patient with bipolar disorder and cocaine abuse who managed to become abstinent from cariprazine. The second and third cases describe patients with psychosis suffering from positive, cognitive and mood symptoms who were non-adherent to previous medication. In both cases, cariprazine was well-tolerated and effective in alleviating symptoms, thus improving their everyday functioning as well. In the discussion, the associations between symptom domains and the receptor profile of cariprazine are also highlighted, providing an explanation of the observed effects. It is concluded that cariprazine is a good treatment option for patients with symptoms of psychosis and addiction; is well-tolerated without the induction of side effects such as weight gain or sedation; and is appropriate for patients who have problems with adherence.

## Introduction

Cariprazine is a third-generation antipsychotic medication with a unique mechanism of action; it is a dopamine D_3_/D_2_ and serotonin 5HT_1A_ partial agonist and serotonin 5HT_2A_ antagonist with preferential binding to the D_3_ receptors (1). Research showed that cariprazine has almost 10-fold greater *in vitro* affinity for the D_3_ receptor than for the D_2_, while *in vivo*, the occupancy of the D_3_ and D_2_ receptors are balanced (2, 3). Cariprazine also has a high affinity for the 5HT_1A_ and 5HT_2B_ receptors and a low affinity for 5HT_2A_, 5HT_2C_, histaminergic, adrenergic, and cholinergic receptors – as seen in Table 1 (4). It is metabolized by the CYP3A4 enzyme into two major active metabolites: desmethyl cariprazine (DCAR) and didesmethyl cariprazine (DDCAR), both pharmacologically equipotent to cariprazine and known to be jointly responsible for the overall therapeutic effect (5). According to the product label (6), the mean concentrations of DDCAR and DCAR relative to cariprazine by the end of a 12-week treatment are 400 and 30%, respectively (Table 2). This unique pharmacokinetic profile and mechanism of action, i.e., the D_3_ affinity combined with the actions of the different 5HT receptors, makes cariprazine capable of alleviating the symptoms of different psychiatric disorders, such as schizophrenia or bipolar I disorder.

**TABLE 1:** Receptor profile and binding affinities of cariprazine.

Receptor	Binding profile	Affinity category	Ki (nM)
Dopamine type 3	Partial agonist	High	0.085
Dopamine type 2L	Partial agonist	High	0.49
Dopamine type 2S	Partial agonist	High	0.69
Serotonin type 2B	Antagonist	High	0.58
Serotonin type 1A	Partial agonist	High	3.0
Serotonin type 2A	Antagonist	Moderate	19.0
Histamine type 1	Antagonist	Moderate	23.0
Serotonin type 7	Antagonist	Low	111.0
Serotonin type 2C	Antagonist	Low	134.0
Alpha type 1A	Antagonist	Low	155
Muscarinic	Antagonist	No appreciable affinity	IC_50_ > 1,000

*Ki, Inhibition Constant; PDSP, Psychoactive Drug Screening Program. All Ki values have been taken from the PDSD Ki database except for alpha type 1A and muscarinic Ki values that are from Citrome 2015 (4).*

**TABLE 2:** Pharmacokinetic proprieties of cariprazine.

Pharmacokinetic	Drug information
Absorption	Following multiple-dose administration, peak plasma concentrations for cariprazine and the major active metabolites occur at approximately 3–8 h post dose
Distribution	Based on a population pharmacokinetic analysis, the apparent volume of distribution (V/F) was 916 L for cariprazine, 475 L for DCAR, and 1,568 L for DDCAR, indicating an extensive distribution of cariprazine and its major active metabolites. Cariprazine and its major active metabolites are highly bound (96–97% for CAR, 94–97% for DCAR, and 92–97% for DDCAR) to plasma proteins.
Metabolism	Cariprazine is metabolized by CYP3A4 and, to a lesser extent, by CYP2D6, to DCAR and HCAR. DCAR is further metabolized by CYP3A4 and to a lesser extent by CYP2D6 to DDCAR and HDCAR. DDCAR is further metabolized to HDDCAR by CYP3A4.
Elimination	Mainly through hepatic metabolism. Following administration of 12.5 mg/day cariprazine to patients with schizophrenia, 20.8% of the dose was excreted in urine as cariprazine and its metabolites. Unchanged cariprazine is excreted by 1.2% of the dose in urine and 3.7% of the dose in feces.

*HCAR, hydroxy cariprazine; HDDCAR, hydroxy didesmethyl cariprazine.*

Indeed, cariprazine is currently approved for the treatment of schizophrenia in adults (1.5–6.0 mg/day), as well as for the treatment of depressive, acute manic, or mixed episodes associated with bipolar I disorder (3.0–6.0 mg/day) by the Food and Drug Administration (FDA). In Europe, the European Medicines Agency (EMA) also approved cariprazine for the treatment of schizophrenia in adults. Moreover, recent results of Phase II clinical trials found support for the notion that cariprazine is also effective for the adjunctive treatment of the major depressive disorder (MDD) (7).

The aim of the current paper was to present three patient cases who have been successfully treated with cariprazine and achieved improvements in both their symptoms and functionality. A short summary of the cases is presented in Table 3. The present report was written following the CARE guidelines (8).

**TABLE 3:** Summary and comparison of the clinical cases.

	Case 1	Case 2	Case 3
Sex	M	M	F
Family history	Psychological disorders	Negative	Depressive disorder
Illness duration	10 years	3 years	3 years
Age at first psychiatric evaluation at AMHS	25 years	35 years	29 years
Diagnosis	Bipolar disorder	Schizophrenia	Schizophrenia
Suicide attempts	Yes	No	No
Drug abuse	Cannabis, LSD, MDMA, heroin, cocaine	No	No
Previous treatments	Valproic acid 1,000 mg/day, quetiapine prolonged release 250 mg/day, and paroxetine 30 mg/day Valproic acid 1,000 mg/day, paroxetine 30 mg/day, bupropion 300 mg/day, and quetiapine prolonged release 600 mg/day	Amitriptyline 10 mg/day and perphenazine 4 mg/day	Olanzapine 5 mg/day up titrated to 10 mg/day Olanzapine 10 mg/day and venlafaxine up to 150 mg Lurasidone up to 148 mg Aripiprazole up to 30 mg Paliperidone up to 9 mg
Dosages of cariprazine	1.5 mg/day increased to 3 mg/day after 7 days	1.5 mg/day increased to 3 mg/day after 7 days	1.5 mg/day increased to 3 mg/day after 6 days and further increased to 4.5 mg/daily after 7 days

## Case 1: Improvement of Dysthymic Disorder and Substance Abuse in a Bipolar Patient

The first case describes a 35-year-old male patient with bipolar disorder who had been followed by the Adult Mental Health Service (AMHS) for about 10 years.

The family history of the patient revealed psychiatric disorders on the paternal side; the patient’s father suffers from bipolar disorder and was hospitalized for anticonservative gestures, while his uncle was diagnosed with schizophrenia. In terms of birth and childhood, the patient was born at full term with vaginal delivery, and during his early school years, he showed sufficient performance with the help of a support teacher. Nonetheless, from the ages of 7–9, he had several serious nightmares from which he could not be awakened. He then began to use drugs such as cannabis, Lysergic acid diethylamide (LSD), and Methylenedioxymethamphetamine (MDMA) at the age of 13. About 4 years later, he switched to heroin and cocaine and was therefore referred to the Addiction Treatment Service. He received treatment with buprenorphine for about 3 years, which allowed him to become drug-free. Due to this episode of substance abuse and difficulties in interpersonal relationships, the patient obtained a high school qualification later than his peers.

In 2012, the patient was presented at the AMHS for the first time due to repeated suicide attempts, for example, *via* ingesting dangerous doses of drugs. He exhibited a dysphoric mood with high levels of anxiety. Nonetheless, he had no serious alterations in thoughts or sensory disturbances, and his drug test was also negative. A treatment with valproic acid (1,000 mg/day), quetiapine prolonged release (250 mg/day), and paroxetine (30 mg/day) was started. Some improvement in affective symptoms was achieved, allowing the patient to work at a mechanical company.

Due to a later relapse of dysthymic disorder, he sought urgent help from the AMHS. His medication regimen was changed (valproic acid 1,000 mg/day, paroxetine 30 mg/day, bupropion 300 mg/day, and quetiapine prolonged release 600 mg/day), but it did not yield sufficient improvements. As self-medication, the patient started to use cocaine again on a weekly basis, hoping that this will allow him to improve his dysthymic symptoms and return to work.

In 2020, cariprazine was offered as an add-on therapy to his existing medication regimen (valproic acid, selective serotonin reuptake Inhibitors, and bupropion), which the patient accepted. Cariprazine was started at 1.5 mg/day for 7 days with a subsequent increase to 3 mg/day. By the third week of cariprazine treatment, the use of cocaine progressively decreased, and then, it was completely terminated. In addition, feelings of anguish and other affective symptoms were also reduced. After 2 months of cariprazine treatment, a state of euthymia was observed with good tolerability and no cocaine consumption.

## Case 2: Alleviating Cognitive Symptoms of Psychosis

The second case is about a 38-year-old male patient with psychosis, keratoconus, and obesity. The patient was born vaginally, but during early childhood, he showed a delay in psychomotor development, for which he was referred to a child neuropsychiatry clinic and was assigned a speech therapist and a support teacher. He had no family history of psychiatric disorders, although his parents might have mild intellectual disability. Until his first visit to the AMHS, he was functioning well with a fair level of autonomy; he had temporary jobs such as gardening, obtained a driving license, and had a group of peers linked to the parish. He never took psychotropic drugs and lived with his elderly parents.

In 2021, the patient presented at the AMHS with various neuropsychiatric symptoms: he had two episodes of short-lived prosopagnosia, initial insomnia, and stopped driving his car due to fear of not being able to find the road. In addition, he also developed difficulties in writing and choosing the right words, stuttering as well as mental confusion. During his hospitalization in the intensive care unit, various examinations, such as magnetic resonance imaging (MRI), electroencephalogram (EEG), and brain computed tomography (CT), were carried out to understand the nature of his symptoms. As all the tests were negative, he was discharged and referred to the outpatient psychiatric clinic.

During his first visit, he appeared to be perplexed, confused, depressed, and anxious. Furthermore, he presented with obsessive ideation with doubts of harm, ideas of reference and influence such as “it seems to me that the television talks about me,” and repetition of fixed phrases and automatisms. Although he had social and cognitive (attention, concentration) difficulties, no alterations in sensation and perception were reported. Due to his symptoms, he was unable to continue working.

The patient received a combination treatment (amitriptyline 10 mg/day and perphenazine 4 mg/day), which was ineffective due to non-adherence. Then, treatment with cariprazine was initiated with 1.5 mg/day and increased to 3.0 mg/day due to good tolerability and a partial response after a week. At the same time, amitriptyline 10 mg/day and perphenazine 4 mg/day was still prescribed. In response to treatment with cariprazine, the patient was more relaxed and less confused with a more fluid and organized speech at the control visit. Further improvements were seen after 1 month; delusional thoughts and concentration difficulties disappeared, sleep became regular, and social skills returned almost to the level of premorbid functioning.

Three months after the initiation of cariprazine treatment, the patient returned to work and remained adherent to the medication, which did not induce any side effects. Importantly, no weight gain occurred with cariprazine, which, given his obesity, would have influenced adherence negatively and would have been harmful to the physical health of the patient.

## Case 3: Achieving Euthymia After Frequent Switching

The third case report describes a 32-years old Albanian female patient who moved to Italy at the age of 18 after finishing high school and getting married, leaving her parents and siblings behind. Although at first, she worked in a manufactory, later she decided to stay at home and focus on the upbringing of her two children. Her family history was positive for depressive disorder, but her personal history was negative.

Before seeking help at the psychiatric unit, she had been maintaining an extramarital relationship for several months and developed delusional beliefs such as that her phone was under the control of her lover and his friends. In addition, she also developed ideas of reference, interpreting promotional messages as personal messages with which her lover tried to communicate.

When she first sought help at the psychiatric unit, she seemed cooperative, attentive, and oriented to time, person, place, and circumstances. She had normal personal hygiene and clothing and could maintain attention and concentration. Her speech was rapid, but appropriate in volume, quantity, and quality. The content of her thoughts was characterized by the abovedescribed delusional beliefs, causing growing feelings of anguish and anxiety. No hallucinations were reported. Her mood was dysphoric, and she reported sleep disturbances. Although her level of awareness and insight was poor, she acknowledged impairments in social and personal functioning.

In 2019, pharmacological treatment with olanzapine 5 mg/day was initiated. After an up-titration to 10 mg/day, a rapid decrease in dysphoria, anxiety, and hyperarousal was detected along with restoration of sleep patterns. Nonetheless, after a few months of treatment, the patient developed a depressed mood which was then addressed with the antidepressant therapy (venlafaxine 150 mg/day). In 4–6 weeks, full remission was achieved.

Six months later, the patient showed up again at the clinic; she stopped taking olanzapine due to side effects (excessive sedation and weight gain), which resulted in the reoccurrence of psychotic symptoms. First, lurasidone (148 mg/day) was prescribed without any success, then aripiprazole (30 mg/day), which was stopped because of the development of akathisia, and finally paliperidone (9 mg/day), which was also terminated due to amenorrhea.

Pharmacological treatment with cariprazine (1.5 mg/day) was initiated in 2021. After 6 days of treatment, the dose was increased to 3.0 mg/day, and after another week, it was further increased to 4.5 mg/day due to good response and no adverse effects. Within a few weeks, the patient achieved remission of psychosis and eventually euthymia, so she started looking for a new job.

## Discussion

The above-described clinical cases provide a detailed insight into the characteristics of cariprazine in terms of both effectiveness and tolerability. They show how cariprazine has the ability to address a range of symptoms from delusions to cognitive disturbances regardless of disorder type and without the induction of metabolic, endocrine, or cardiovascular side effects that are common with other antipsychotic medications.

For instance, the first case of the series describes the effectiveness of cariprazine in a bipolar disorder patient with symptoms of mood and addiction. The efficacy of cariprazine in bipolar depression was established in three short-term, double-blind, placebo-controlled, randomized Phase II/III clinical trials where the change from baseline to week 6 on the Montgomery Asberg Depression Rating Scale (MADRS) was significantly greater in patients treated with cariprazine (1.5–3.0 mg/day) compared with patients on placebo (9–11). The effectiveness of cariprazine in mood is attributed to the fact that it acts as a partial agonist at the presynaptic D_3_ receptors in the ventral tegmental area (12) as well as it has a relatively high affinity for the 5HT_1A_ receptors that are related to the antidepressant effect (13). Importantly, D_3_ receptors might also be involved in substance abuse as they are highly expressed in the reward circuitry of the limbic system, which is responsible for motivation and emotions (14, 15). Indeed, results from preclinical studies indicate that D_3_/D_2_ partial agonists might be effective in preventing relapse of cocaine abuse (16) and that 5HT_2B_ antagonists may contribute to the prevention of MDMA relapse (17). In line with these preclinical studies, other cases have also shown the benefits of cariprazine in the treatment of bipolar disorder with substance abuse (18, 19). Similar to the present case, Sanders and Miller described a 51-year-old male bipolar disorder patient with alcohol use and cocaine craving, who became abstinent with cariprazine monotherapy (18). In addition, there are two running clinical trials that aim to examine the efficacy of cariprazine in substance abuse disorder, indicating that there is a definite potential for using cariprazine in the field of addiction (20, 21).

The other cases describe patients with different psychotic symptoms; while in the case of the second patient, cognitive disturbances and mood symptoms were prevalent, the third patient was suffering more from positive symptoms, mainly delusional thoughts. Regarding the latter, the efficacy of cariprazine in acute schizophrenia was studied in three short-term, double-blind, placebo-controlled, Phase II/III clinical trials where the primary outcome measure was mean change from baseline to week 6 on the Positive and Negative Syndrome Scale (PANSS) (22–24). Importantly, pooled results of these trials indicated statistically significant differences versus placebo on all 5 PANSS factors: positive, negative, cognitive, anxiety/depression, and hostility/excitement (25). These results can be explained again by the unique receptor profile of cariprazine. The high affinity for the D_3_ receptor seems to be responsible for the effect on the negative, cognitive, and anxiety/depression symptoms, while the D_2_ for the positive and hostility/excitement symptoms (4). The other receptor affinities such as serotonergic, histaminergic, adrenergic, and cholinergic receptor affinities also account for the effectiveness on negative and cognitive symptoms as well as for the favorable safety profile (4). Indeed, the incidence of sedation, hyperprolactinemia, and metabolic side effects are low with cariprazine (26) which has been also highlighted in the second patient as a definite advantage, given that he had already been suffering from obesity and further weight gain would have worsened his physical health. The absence of weight gain was important for the third patient as well, as previously this was one of the reasons why she stopped her treatment with olanzapine. Although the most common side effect of cariprazine is akathisia (26), it has not been reported by any of the patients. In fact, in the third patient case, aripiprazole, another partial agonist, was terminated due to akathisia, whereas no such incidence happened when the patient switched to cariprazine. Finally, choosing cariprazine as a treatment for the second patient was also supported not only by the fact that cariprazine is a well-tolerated medication but also because it has a long half-life (27). This was important given the fact that this patient showed poor adherence previously.

It is also important to note that the described patients also experienced functional improvement. Being able to return to work is often less emphasized than symptomatic improvements even though it is a clear indication of the patient’s level of functioning. *Post hoc* analyses of clinical trials and other reviews also suggest that cariprazine has the ability to improve everyday functioning in patients with schizophrenia (28, 29) and bipolar disorder (30).

To conclude, the pharmacokinetic and pharmacodynamic characteristics of cariprazine make this third-generation antipsychotic medication a first-line treatment option in patients with symptoms of psychosis, addiction, and mood, who also displayed poor adherence to treatment as well as high metabolic and cardiovascular risks. Cariprazine provides a solution to all these aspects, allowing the patients to achieve full remission and functional improvement.

## Data Availability Statement

The original contributions presented in this study are included in the article/supplementary material, further inquiries can be directed to the corresponding author.

## Ethics Statement

Written informed consent was obtained from the individual(s) for the publication of any potentially identifiable images or data included in this article.

## Author Contributions

All authors listed have made a substantial, direct, and intellectual contribution to the work, and approved it for publication.

## Conflict of Interest

The authors declare that the research was conducted in the absence of any commercial or financial relationships that could be construed as a potential conflict of interest.

## Publisher’s Note

All claims expressed in this article are solely those of the authors and do not necessarily represent those of their affiliated organizations, or those of the publisher, the editors and the reviewers. Any product that may be evaluated in this article, or claim that may be made by its manufacturer, is not guaranteed or endorsed by the publisher.
